# Bio-Based Binder Development for Lithium-Ion Batteries

**DOI:** 10.3390/ma16165553

**Published:** 2023-08-10

**Authors:** Illia Dobryden, Céline Montanari, Dhrubajyoti Bhattacharjya, Juhanes Aydin, Anwar Ahniyaz

**Affiliations:** RISE Research Institutes of Sweden, Drottning Kristinas väg 61, 114 28 Stockholm, Sweden; celine.montanari@ri.se (C.M.);

**Keywords:** sustainable, battery, binder, anode, cathode

## Abstract

The development of rechargeable lithium-ion battery (LIB) technology has facilitated the shift toward electric vehicles and grid storage solutions. This technology is currently undergoing significant development to meet industrial applications for portable electronics and provide our society with “greener” electricity. The large increase in LIB production following the growing demand from the automotive sector has led to the establishment of gigafactories worldwide, thus increasing the substantial consumption of fossil-based and non-sustainable materials, such as polyvinylidene fluoride and/or styrene-butadiene rubber as binders in cathode and anode formulations. Furthermore, the use of raw resources, such as Li, Ni, and Mn in cathode active materials and graphite and nanosilicon in anodes, necessitates further efforts to enhance battery efficiency. To foster a global sustainable transition in LIB manufacturing and reduce reliance on non-sustainable materials, the implementation of bio-based binder solutions for electrodes in LIBs is crucial. Bio-based binders such as cellulose, lignin, alginate, gums, starch, and others can address environmental concerns and can enhance LIBs’ performance. This review aims to provide an overview of the current progress in the development and application of bio-based binders for LIB electrode manufacturing, highlighting their significance toward sustainable development.

## 1. Introduction

The rechargeable battery revolution has led to substantial advancements in global and daily-life-applied electrification, since the first developed rechargeable lead-acid battery in 1860 by Gaston Planté [1]. Rechargeable batteries are key components in modern portable electronics, including cell phones, laptop computers, and remote digital systems (cameras, radios, music players, sensors, controls, etc.). Rechargeable lithium-ion batteries (LIBs) are particularly significant for their high-energy density, long lifespan, and efficiency, making them crucial in the sustainable transition to electric vehicles (EVs) with energy densities of up to 500 Wh L^−1^ and specific capacities of 350 Wh kg^−1^, enabling driving distances beyond 500 km [2,3]. LIB technology is also suitable for grid storage solutions, minimizing carbon dioxide (CO_2_) emissions and providing fossil-free energy sources once in service. The invention and development of the LIB began in the 19th Century with the discovery of lithium (Li), the most-reactive metal with the lowest atomic number (6.95 atomic weight) and density (0.534 g/cm^3^), by Johan August Arfwedson and later reported by Jöns Jakob Berzelius in 1818 [1]. Later, William Thomas Brande successfully isolated elemental Li and provided its material description in 1821 [1]. It took several years until the development and commercialization of the first non-rechargeable Li battery in a non-aqueous electrolyte in the 1970s [4]. The first rechargeable Li metal anode battery was developed using the MoS_2_ cathode material in an AA cell design by Moli Energy in 1985, but it resulted in many fire incidents [5]. Later, in 1986, the Asahi team developed the first LIB configuration close to modern LIBs based on a LiCoO_2_ cathode, a non-graphitic anode, a polyolefin separator, and a lithium perchlorate (LiClO_4_) electrolyte [6]. This type of LIB was further commercialized by Sony in the 1990s with a capacity of more than 4 V [7]. Since the development of modern rechargeable LIBs, their performance requirements and production volumes have dramatically increased driven by the rapid advancements and commercialization of EV and power grid storage technology. For instance, 1 TWh of LIB capacity can only provide 10 million EVs, which is about 10% of the annual global vehicle production [3]. To meet this demand and the increase in LIB production, significant amounts of fossil and non-sustainable materials such as polyvinylidene fluoride (PVDF) and/or styrene-butadiene rubber (SBR) are consumed as binders in cathode and anode formulations associated with toxic organic solvents [8]. Additionally, the increased LIB production in the past decades has impacted the availability of raw resources such as Li, Ni, and Mn used in cathode active materials. The predicted resource shortage for LIB production will further drive the introduction of novel and next-generation cathode and anode materials [3].

A typical LIB consists of an anode (negative electrode), a cathode (positive electrode), a separator (of optimized wettability toward an electrolyte and to separate the anode and cathode to prevent thermal runaway and short-circuiting), and an electrolyte (to promote the transfer of the Li ions between the cathode and anode during the charge/discharge process) [9]. The overall LIB performance is strongly affected by the electrode materials and their architecture. The optimization work of electrodes and their composition has been under a large research focus [2,3,9,10,11]. Electrodes are composed of active materials, conductive additives, polymeric binders, and metal current collectors. Metal foils of aluminum and copper are commonly used as current collectors for LIBs because of their high electrical conductivities. Polymeric binders are used in both electrodes to promote the cohesion of particles in the electrode. Binders also ensure a favorable viscosity of the slurry for uniform coating and adhesion to the metal current collector [8,12,13]. Moreover, binders facilitate the Li-ion transfer and have an impact on the electrochemical stability and specific capacity of the batteries [8]. The role and importance of binders in LIBs is becoming more relevant as new technologies for the next-generation anodes, e.g., based on the implementation of nanosilicon, are being developed.

Binder selection, concentration, and composition often differ from cathode to anode formulations in LIBs. Binders in LIBs are classified into three categories, organic solvent-soluble, water-soluble, and binderless systems. The most-commonly used solvent-soluble binder is PVDF, which provides excellent electrochemical stability, binding capacity, and overall efficiency. Other often-studied systems are poly(acrylic acid) (PAA), poly(vinyl alcohol) (PVA), and polyacrylonitrile (PAN), which in contrast to PVDF can facilitate Li-ions’ migration into graphite, provide additional binding strength improvement due to the presence of hydroxyl groups, and affect the formation of the solid electrolyte interphase (SEI) [14,15]. For instance, the PAN polymer enables the formation of nitrile groups that demonstrate the improved transfer of Li ions and increase active material contact efficiency [16]. Further investigations are being carried out to explore material design strategies for novel binder systems in the next-generation LIBs. These strategies typically involve the development of multifunctional and hybrid binder systems to introduce a passivation layer and enhance both ionic and electronic conductivity [17,18,19,20].

Water-based binder systems offer opportunities for sustainable development. The most used water-based binder systems, especially in the case of the anode for LIBs, are SBR or a hybrid system of SBR mixture with carboxymethyl cellulose (CMC) [21] and acrylonitrile binders [22]. Water-soluble binders open great prospects for the introduction of environmentally friendly bio-based solutions, such as cellulose, natural polylactides, alginates, lignin, etc. The implementation of bio-based binders for LIBs is currently under strong research focus and industrial interest because of the combination of the low environmental impact, the ability to improve electrode processing, and the versatile functional property range that they offer. The selection of bio-based binders is broad and offers unique surface functionality with available functional and polar surface groups, such as carboxylic acid and hydroxyl groups. Thus, various binding mechanisms and resulting interfacial strength can be achieved via the introduction of van der Waals, hydrogen, and covalent bond interactions [8]. This can facilitate, for instance, a self-healing process in LIB next-generation anodes containing Si via hydrogen bond interactions [23].

With the rapid development of the scale at which batteries are being manufactured and the emergence of gigafactories for LIB production, the cost, processability, and sustainability of electrode materials are key considerations. For commercial applications, new binder materials should be inexpensive, environmentally friendly, and processable in non-toxic solvents. In this review, the recent developments in bio-based binders for both cathodes and anodes in the LIB technology are described for the development of more-sustainable battery materials.

## 2. Binder Function and Mechanisms

**Figure 1:**
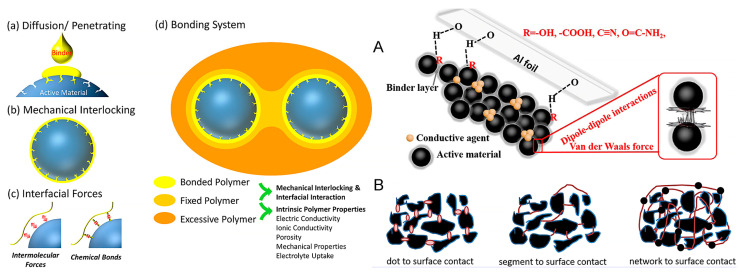
Schematics of the mechanical interlocking and interfacial force binding mechanism are illustrated in the image. The classification of the chemical binding such as dot-to-surface contact, segment-to-surface contact, and network-to-surface contact is shown to the right. (**A**) Illustrates the schematic diagram of chemical connection and (**B**) the schematic demonstration of the interactions. The image to the left is reproduced with permission from [24], and the image to the right is reproduced with permission from [8].

The binder is a critical component in both anode and cathode electrodes both for the electrochemical performance of the battery and the production process. The binder is a polymer that offers strong adhesion to the active materials (e.g., graphite), carbon additive (e.g., carbon black), and metal current collector (e.g., copper foil). The binder is applied during the electrode’s fabrication, where, typically, an electrode slurry is produced by mixing the anode materials with the binder dissolved in a solvent, which is then cast onto the current collector foil. The binder enables the cohesion and binding of the anode particles together and also increases the viscosity of the slurry to ensure uniform and smooth coating on the current collector during the electrode fabrication process. The good adhesion of the active materials to the current collector is critical to the electrochemical properties; thus, the binder directly affects the battery’s performance. Therefore, the binder should also exhibit good electrochemical stability and high ionic conductivity. The driving force and mechanism for a binder to bind surfaces can be either mechanical interlocking, interfacial binding forces, or a combination of both [8,24,25,26]. These binding mechanisms are illustrated in Figure 1. Mechanical interlocking is based on the diffusion of a binder into the surface defects (e.g., pores) and then interlocking the surfaces due to the reaction mechanism of the binder system, such as drying or polymerization [8,24]. The mechanical interlocking strength is strongly affected by the surface roughness and porosity of the active material. The interfacial binding force mechanism is one of the most-common approaches to introducing binders in electrode materials. The interaction forces such as van der Waals, hydrogen bonding, covalent bonding, and coordinate bonding promote the binding strength.

Various binder systems have different functional groups available that can bind with the unoccupied orbital of -H in the active material and current collectors [8,24]. Moreover, the strength of the binding can be tuned by adjusting the functional groups of a binder and their density. One approach to classifying the chemical binding is via the introduction of dot-to-surface contact, segment-to-surface contact, and network-to-surface contact [8], as illustrated in Figure 1. Most common LIB electrode linear binders such as PVDF and CMC can be attributed to segment-to-surface contact, whereas chemically driven reactions, such as cross-linking, are required for network-to-surface contact [8].

**Figure 2:**
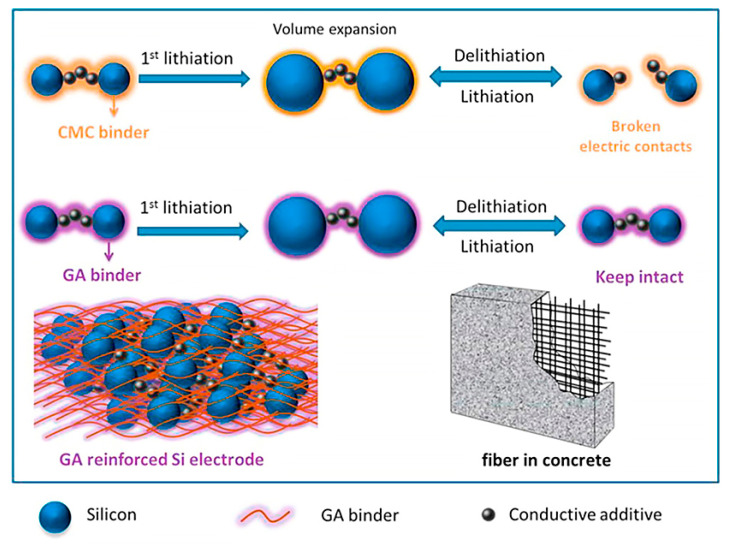
Schematics of the improved mechanical integrity of next-generation Si-based anodes via the introduction of a reinforcement binder are illustrated. The image is reproduced with permission from [30].

**Figure 3:**
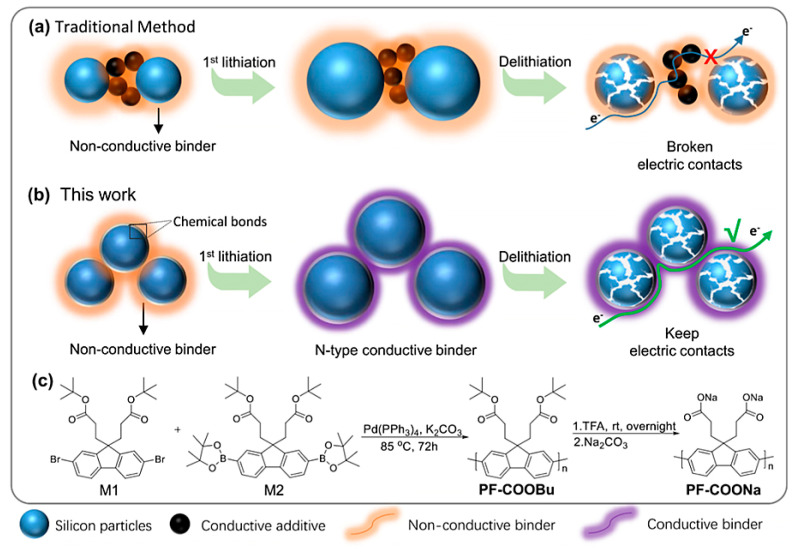
Schematics illustrating the preservation of conductive network during delithiation/lithiation cycling in active material via the introduction of conductive binders. Traditional and conductive binder approaches to address volume expansion are demonstrated in (**a**,**b**). Synthetic scheme of the conductive polymer is illustrated in (**c**). The image is reproduced with permission from [20].

In the case of a next-generation anode active material based on nanosilicon technology, a binder system has also another functionality dealing with nanosilicon expansion during the delithiation and lithiation cycles [20,24,27,28]. Common graphitic anodes have a limited specific capacity of 372 mAh g^−1^, whereas the next-generation technology based on the introduction of silicon (Si) can significantly increase the theoretical specific capacity to 4200 mAh g^−1^ [11,27,29]. However, silicon-based anodes experience a large volume expansion of up to 300% during delithiation/lithiation cycles due to Li-ion insertion and extraction from Si [11,29]. Such a significant volume change causes a dramatic effect on the anode’s performance due to the delamination of the anode coating from the current collector and destruction of the conductive network in the active material [27]. Thus, a critical role of a suitable binder system is in improving the mechanical integrity of the active next-generation anode material and keeping the initial anode performance for long-life battery cycling. Moreover, the introduction of novel conductive polymer binders is another strategy to prevent the degradation of Si-based anode performance by promoting a stretchable conductive network [20,27]. The schematics of Si-based anodes’ reinforcement and facilitation of a stable conductive network with suitable binders are shown in Figure 2 and Figure 3.

## 3. Bio-Based Binder Development for Anodes in LIBs

**Figure 4:**
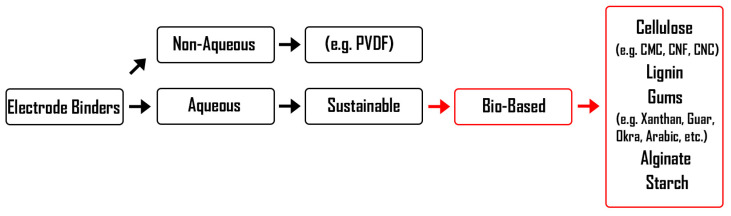
A sketch demonstrating the bio-based binder class focus of this review, including cellulose, lignin, various gums, sodium alginate, and starch binders for the preparation of LIB anode and cathode slurries.

The development of novel bio-based binder systems in the LIB industry is emerging to meet the current requirements of sustainability and improved battery performance to optimize resource usage. In particular, the use of binders soluble in aqueous-based systems is of high interest and importance for sustainable development [21,24,26,31,32,33]. Due to health concerns and in line with the impending ban of perfluoroalkyl and polyfluoroalkyl substances (PFAS) in the European Union (as stated by ECHA’s Committee for Risk Assessment (RAC), ECHA/NR/23/10 and ECHA/NR/22/05), the development of environmentally friendly and non-toxic binder alternatives becomes essential for the near future. Water-soluble slurries are favorable to the introduction of environmentally friendly bio-based binders, such as cellulose [21,33,34,35,36,37], gums [30,38,39,40,41,42,43], starch [44,45,46], lignin [28,36,47], alginates [48,49], etc. [50,51,52,53,54,55,56]. The implementation of bio-based binders for LIBs offers a low environmental impact, a proven ability to improve electrode processing, and versatile functional properties with available functional and polar surface groups, such as carboxylic acid and hydroxyl groups. The bio-based binder class for anodes and cathodes, which is part of a broader sustainable polymer binder area, is the main focus of this review work, as illustrated in Figure 4.

The manufactured electrode performance is usually evaluated via measured parameters such as specific capacity (in mAh g^−1^), the Coulombic efficiency (CE, in %), charging/discharging rate, cyclability, capacity retention (in %), etc. Coulombic efficiency evaluates the ratio of lithium charging capacity to the discharging capacity and is a measure of the electrochemical energy-storing reaction’s reversibility. Moreover, for the first cycle CE, a lower efficiency is usually observed for cathode and anode materials because of a change in the material cathode structure during delithiation [57] and the formation of the SEI layer in the anode material [11,58]. There are also other origins of Coulombic efficiency loss [59]. Interestingly, SEI layer formation can be tuned by the binder selection toward improved cycling performance [12]. The formation of a uniform and stable SEI layer due to binder properties was shown to improve the first cycle CE and reversible capacity for over 50 cycles [60,61]. Thus, for new bio-based binder developments in LIB electrodes, it is important to evaluate the first cycle CE and the effect of binders on the formation of a stable SEI layer. The build-up of the SEI layer and the water content changes using aqueous binders and their effect on the battery performance were studied using X-ray photoelectron spectroscopy [62].

**Cellulose** is the most-abundant biopolymer on Earth, exhibiting renewable, degradable, biocompatible, and cost-efficient properties. Cellulosic binders are available in various forms, from cellulose nanofibers (CNFs) of a high aspect ratio, cellulose nanocrystals (CNCs), microparticles of carboxymethyl cellulose (CMC), and microscale pulp fibers. In the LIB applications, CMC (a derivative of cellulose with carboxymethyl groups of varying degrees of substitution), as well as nanocelluloses including CNFs have been reported as binders. CMC has shown promising results and is currently used commercially in anode formulations because of its low cost and sustainability compared to PVDF and its favorable solubility in water. Moreover, the surface chemistry of CMC decorated with carboxymethyl groups provides a favorable chemical bonding with the graphite active material. Although CMC is performant as a binder and provides shear-thinning behavior to the slurry [63], it possesses high stiffness and a small fracturing strain, which can affect the overall mechanical properties and long-term performance. Hence, CMC is commonly combined with a rubber (typically SBR) to optimize the mechanical properties of the slurry coated on the current collector. In particular, Na-CMC combined with fossil-based SBR copolymer provides a cost-efficient binder solution of anodes in LIBs [34]. It has been demonstrated that, in comparison with other common binders, a CMC-based binder solution in LIB Si-based anodes can provide a specific capacity of about 1153 mAh g^−1^ for 35 cycles [23] and 1100 mAh g^−1^ for 70 cycles [64]. The cyclic performance of an active mass loading of 2.5 mg Si cm^−2^ using the CMC binder with a binder content from 4 to 8 wt% also demonstrated a high capacity of 1000 mAh g^−1^ (4 wt% binder) to 1900 mAh g^−1^ (8 wt% binder) for 50 cycles [65]. Furthermore, CMC promotes a specific bonding between the carboxylic groups and the OH groups on the Si oxide surface. Another study has demonstrated a specific capacity of 1544 mAh g^−1^ after 100 cycles when CMC binder was used [28]. The initial CE was 85.3% with a further gradual increase after the first cycles. In the case of common graphite electrodes, CMC combined with SBR and crosslinked poly(acrylic acid) (PAA) provides a stable specific capacity of more than 300 mAh g^−1^ for the first 35 cycles [60]. The capacity retention was reported to be 96.6% at 0.5 C over 35 cycles in the case of a coin half-cell test. It was also found that such a binder combination (CMC/SBR/PAA) is very efficient for a thick graphite anode and enhances electrochemical performance. Sulfobetaine methacrylate modification of carboxymethyl cellulose has also demonstrated improved binding properties and a specific capacity of 141 mAh g^−1^ at a 0.1 C rate using a graphite anode in LiFePO_4_ [37] in comparison to 108 mAh g^−1^ for pure CMC. The SEI layer was formed during the first three cycles at a 0.1 rate, and the discharged capacity loss was 12.2% for CMC binder and 6.6% for carboxymethyl cellulose with sulfobetaine methacrylate modification. The first step CE for both binders was 75% (CMC) and 83% with a gradual improvement to about 90 and almost 100% after three cycles. The limitations of using CMC as a binder in LIB anodes are the possibilities for trapping electrons within the carbon coating and decreasing the electronic conductivity and possible decrease of Coulombic efficiency due to the formation of -CH_2_COOLi groups [32,66]. Furthermore, the binder decomposition can contribute to the formation of a solid electrolyte interphase layer. In the context of sustainable development and health safety [67], the required use of fossil-based SBR in combination with CMC is also a limitation that remains to be addressed toward fully bio-based binders for anode slurries. The CMC and Na-CMC binder effect on the anode slurry’s rheological properties and interaction with graphite and carbon black was studied in the literature [68,69]. The Na-CMC binder has also demonstrated good coating properties and a good performance of about 300 mAh g^−1^ at 0.1 C at a binder content of 10 wt% [70]. The first cycle CE was about 90% with a further increase to above 99.5%, which can be attributed to the formation of the SEI layer [62,70]. The carboxymethyl cellulose lithium binder synthesized using a weak acid was also evaluated and compared to the Na-CMC binder [71,72].

Bio-based CNFs are an interesting binder candidate for aqueous-based anode slurry preparation as their high aspect ratio enables the formation of a strong web-like network around the graphite active material for freestanding and flexible anodes [73,74]. Modified cellulosic fibers, particularly those with aldehyde and carboxyl functionalities [75], hold promise as effective binders for graphite anodes in LIBs, offering both improved electrochemical performance and mechanical strength. In particular, TEMPO–periodate-oxidized cellulose electrodes showed higher specific capacities at high cycling rates (10% increase at 400 mA/g), because of the enhanced chemical compatibility with both graphite and the electrolyte, as well as an ability to form a strong fibrillar network that prevents structural damage upon graphite expansion [76]. Additionally, a sustainable approach was reported to produce freestanding CNF–graphite hybrids (90 wt% graphite) exhibiting energy storage performance (330 mAh/g) and processing speed on par with commercial graphite anodes [77]. These eco-friendly electrodes possess the remarkable ability to be completely recycled, reformed, and reused without compromising their original performance. TEMPO-oxidized CNFs as a binder demonstrated excellent electrochemical performance with a specific capacity of graphite electrodes of 345 mAh/g at C/10, comparable to PVDF graphite anodes (350 mAh/g) [73]. TEMPO-oxidized CNFs have also been used in combination with CMC for the stabilization of Si-anodes in LIBs, where the CNFs act both as the reinforcing material and binder additive [73].

**Lignin**, another biopolymer present in woody biomass, is currently experiencing a high research interest in many relevant areas, as well as various industrial applications. Recent publications in the field of bio-based binders for LIBs have also demonstrated that lignin offers good applicability and has a positive impact on electrochemical performance when introduced to anodes [28,36,47,78,79]. Lignin is a macromolecule, and its mechanical properties need to be optimized for a binder implementation [68]. The usual strategy is using plasticizers such as polyethylene glycol (PEG) [47], grafting, for instance with poly(acrylic acid) [28], and other synthesis routines [80]. In the case of the LiFePO_4_ cathode material and the anode material consisting of graphite, it has been shown that the lignin content needs to be increased up to 8 wt%, and a stable specific capacity of 305 mAh g^−1^ was achieved [47] at 0.1 C with a Coulombic efficiency of above 99% for the first 10 cycles. The specific capacity was shown to decrease to 160 mAh g^−1^ at a higher rate of 1 C. The CE was about 84% in the first cycle and increased above 98% after four cycles. The stability was demonstrated to be good at a C/4 rate for 50 cycles [47]. Grafting lignin with polyacrylic acid and copolymeric binders has also demonstrated favorable applicability for the next-generation anode containing Si-nanoparticles [28]. A stable specific capacity limited at 800 mAh g^−1^ remained for over 940 cycles for the silicon microparticle anode with a maximum reached specific capacity of 1914 mAh g^−1^ [28]. The initial CE was 91% with a gradual increase after the first cycles. In the case of silicon-/graphite-based electrodes, the implementation of a grafted lignin binder resulted in a specific capacity of 492 mAh g^−1^, which remained stable for 100 cycles [28]. Lignin (L) binder modification with polyacrylic acid (PAA) copolymeric binders (L-co-PAA) has also demonstrated promising results [81]. It has been shown that a stable specific capacity of 939 mAh g^−1^ was obtained for more than 1000 cycles using the silicon-based electrodes [81].

Various **polysaccharides**, such as guar gum [38,39,82], gum arabic [30], okra gum [40], xanthan gum [41], tragacanth gum [42], carrageenan [55], etc. [53,83,84,85], are often applied as a binder for LIB anodes including current and next-generation technologies utilizing Si. The gums offer good mechanical property flexibility, available hydroxyl groups, promoting chemical bonding with Si nanoparticles, and often, improved ion conductivity. It has been demonstrated that the introduction of guar gum led to better mechanical and viscosity properties. Furthermore, a specific capacity of 2222 mAh g^−1^ was obtained after 100 cycles [82]. The initial CE was 88.3%, and the maximum reached 99.5% after 100 cycles. The specific capacity of 1000 mAh g^−1^ remained over 930 cycles for Si-nanoparticle-based anodes [82]. Modification of guar gum with hydrogenated carboxyl nitrile rubber has demonstrated improved flexibility to accommodate Si volume expansion and improved electrochemical performance for Si-based anodes in LIBs [39]. A performance with a specific capacity of 1402 mAh g^−1^ for 500 cycles at 800 mA g^−1^ and 1128 mAh g^−1^ at 6000 mA g^−1^ was reported. The good electrochemical performance was attributed to the formed covalent and hydrogen bonds between the binder and Si particles [39]. Introducing guar gum into graphite-based anodes in LIB has demonstrated a stable specific capacity of 310 mAh g^−1^ and a capacity retention of 96% for 50 cycles at a C/10 rate [84]. Enhanced mechanical and electrochemical efficiencies were also observed in the case of a xanthan gum binder for LIBs [41,66]. The specific capacity was found to be relatively stable for the graphite anode and was near 250 mAh g^−1^ after 180 cycles [41]. The initial CE was 91.2%. Furthermore, graphite-based electrodes demonstrated a favorable and stable rate capability when the specific capacity remained above 300 even at a 2 C rate [41]. The implementation of xanthan gum as a binder on a Si-based graphene anode has also demonstrated an improved specific capacity of 725 mAh g^−1^ for 50 cycles [85] at 400 mA g^−1^. Gum arabic and okra gum have also demonstrated good applicability to next-generation Si-based anodes. A specific capacity of above 2000 mAh g^−1^ for 300 cycles was achieved using gum arabic binder at a C/10 rate [30], while the initial CE was below 90%. Limiting the specific capacity at 1000 mAh g^−1^ demonstrated improved long-term stability for 1000 cycles at 1 C [30]. The application of gum arabic binder in a graphite-based anode has also demonstrated a good performance, and a specific capacity above 200 mAh g^−1^ was obtained for 50 cycles at a 0.1 C rate [84]. A stable high specific capacity of 1434 mAh g^−1^ and a CE of 99% for 50 cycles at a rate of 0.1 C was achieved for a Si-based anode using okra-extracted gum [40]. Carrageenan is another natural polysaccharide, usually extracted from seaweeds, with available hydroxyl and sulfonyl surface groups of high suitability for strong hydrogen bonding with Si and improving ion conductivity [84]. The specific capacity was measured around 2031 mAh g^−1^ after 100 cycles for the Si-based anode at a rate of 0.5 C [55]. The reversible capacity was found to be around 1623 mAh g^−1^ with a capacity retention of 51.49% after 300 cycles [55]. In the case of graphite-based anodes, the specific capacity using a carrageenan-based binder was found to be around 300 mAh g^−1^ for the first 20 cycles at 0.1 C [84].

**Sodium alginate** is another natural and linear polysaccharide with surface carboxyl groups, which is derived from, e.g., algae biomass. Sodium alginate has been investigated as a water-soluble binder in the literature; see [48,49,86]. The implementation of sodium alginate as a binder in a graphite-based anode has demonstrated a stable specific capacity of about 300 mAh g^−1^ for about 300 cycles at a rate of 0.1 C [84]. In Si-based anodes, the addition of sodium alginate has also demonstrated an improved and stable specific capacity. A Si–graphene-based anode with a sodium alginate binder provided a specific capacity of 780 mAh g^−1^ after 50 cycles with a retention capacity of about 43.8% of initial performance. Grafting and cross-linking of alginate with polyacrylic acid have improved the stability and electrochemical efficiency of graphite–silicon anodes [49]. A stable specific capacity of 849 mAh g^−1^ for 100 cycles at a 0.1 C rate was achieved. It was also demonstrated that pristine alginate and only grafted alginate without cross-linking did not provide a high stable capacity [49]. The initial CE was improved from 68.9% to 72.8% for the cross-linked alginate binder. In another study, the specific capacity was found to decrease to around 1000 mAh g^−1^ for 200 cycles at a1000 mA g^−1^ rate for a Si-based anode [54].

**Starch** is another attractive water-based binder for LIBs due to its low price and high biodegradability. Furthermore, starch functional surface groups can be tuned via various modification processes [44,45,46,50]. The introduction of starch into silicon-based anodes provided discharge capacity values of below 500 mAh g^−1^ after 100 cycles, whereas fluorinated starch has demonstrated a more-stable and significantly improved capacity of 1864–2874 mAh g^−1^ after 100 cycles [46]. The discharge capacity of around 600 mAh g^−1^ after 200 cycles was achieved using fluorinated starch as a binder in Si–graphite anodes [46]. Starch modification with polyethylene glycol has also demonstrated favorable applicability for Si-based anodes, and a specific capacity of about 1100 mAh g^−1^ was reached after 300 cycles at a CE of 99.9% [45]. The initial CE for pure starch was only 59%, whereas the modified starch’s initial CE was around 85%. These results were reported to be better than for the CMC-, starch-, and PVDF-based systems [45]. Cross-linking starch with maleic anhydride was reported to improve the charge capacity to about 2106 mAh g^−1^ after 200 cycles, whereas uncross-linked starch demonstrated a gradual decrease of capacity below 500 mAh g^−1^ after 500 cycles for Si-based anodes [50]. Starch oxidation contained oxidized amylose, and amylopectin was also proposed as a binder system for Si-based anodes in LIBs, for which a specific capacity of about 2000 mAh g^−1^ for 120 cycles was reached [44]. The development and implementation of new bio-based binders for LIBs is not limited to this review, and new results are reported continuously.

The summary of the specific capacity values for the investigated anode and cathode materials using various bio-based binders is shown in Table 1.

## 4. Bio-Based Binder Development for Cathodes

The development of bio-based binders for the positive electrode, i.e., the cathode, in LIBs is more scarcely reported than for graphite- and Si-based anodes. The binder requirements and performance are more complex due to various applied cathode chemistries, such as LiFePO_4_ (LFP), LiMnO_2_ (LMO), and Li(Ni_x_Mn_y_Co_z_)O_2_ for (x + y + z = 1) (NMC) [2,87]. Moreover, certain cathode materials, especially NMC and LMO, are highly sensitive to humid air and water, and their exposure can result in the formation of lithium carbonates and lithium hydroxide, which can affect the cyclic performance [91]. Several bio-based binder systems have been identified and investigated, such as lignin [47,87,88], sodium alginate [48,86], cellulose-based [89,90,92,93], tragacanth gum [43], and others [94]. It was shown that the modification of the pH in an LFP–CMC-based aqueous slurry can significantly reduce the oxidation of Fe(II) and the formation of Li_3_PO_4_ on the electrode’s surface [95,96]. The use of the lignin binder in the LiFePO_4_ cathode has demonstrated a reversible capacity of 148 mAh g^−1^ at 0.1 C and 117 mAh g^−1^ at a rate of 1 C [47]. The introduction of a lignin-based binder to the NMC111 cathode has demonstrated similar performance to the PVDF binder and a specific capacity of about 140 mAh g^−1^ at 0.1 C [87]. The increase of the C rate led to a significant decrease in the specific capacity for the lignin-based binder system [87]. A more-stable performance was found with the CMC/lignin binder in the NMC111 cathode system at increasing C rates [88]. Both binder systems consisting of CMC/lignin and lignin/water demonstrated about a 140 mAh g^−1^ specific capacity. It was also demonstrated that wetting of NMC111 with the lignin/water binder improved the specific capacity measured over 100 cycles at a 0.1 C rate, and the performance was similar to the PVDF-/NMP-based system [88]. CMC is also used as a binder in LiNi_0.4_Mn_1.6_O_4_ [92] and Li [Li0_.2_Mn_0.56_Ni_0.16_Co_0.08_]O_2_ [89] cathode chemistries. A discharge capacity of 230 mAh g^−1^ after 50 cycles at 0.2 C and 169.5 mAh g^−1^ after 200 cycles at 1 C was found [89]. The performance was similar to the PVDF binder system and proved the applicability of the CMC binder for high-voltage cathode materials. Another study has demonstrated a slight increase of the discharge capacity to more than 120 mAh g^−1^ after 100 cycles at 1 C when a CMC binder was used [92]. It has also been demonstrated that a carboxymethyl chitosan/poly(ethylene oxide) binder can be implemented for the high-voltage LiNi_0.5_Mn_1.5_O_4_ cathode with the reached specific capacity of above 120 mAh g^−1^ at 0.2 C and 0.5 C rates for initial cycles [90]. It has been recently demonstrated that tragacanth gum (TG) can also be used as a suitable aqueous binder for a high-voltage LNMO cathode [43]. The binder content was 3 wt%, and specific capacity values of 122 mAh g^−1^ at 0.1 C and 112 mAh g^−1^ at 1 C were reported. At a very high C rate of 15, the specific capacity was 72 mAh g^−1^, demonstrating a better performance than for the PVDF binder case. The capacity retention was reported to be 41% using the TG binder after 1000 cycles [43]. A sodium alginate binder was also reported to be suitable and improved the performance of LIBs [48,86]. The specific capacity of 200 mAh g^−1^ after 100 cycles at a 0.1 C rate was reported for a Li_2_TP cathode using the sodium alginate binder. The system was found to be stable for over 1000 cycles at 1 C with a measured capacity of 130 mAh g^−1^ and nearly 100% Coulombic efficiency [48].

## 5. Conclusions and Future Perspective

The implementation of bio-based binders in LIB electrodes has shown significant progress in recent studies. The focus is on performance enhancement and the replacement of traditional binders with bio-based alternatives. Various bio-based systems such as cellulose, lignin, gums, alginates, and starches have been investigated as alternative binders to traditional ones such as PVDF and SBR. While the use of raw bio-based materials as binders results in difficulties in achieving stable and long-performing electrodes, additional surface modification and functional group optimization promote bonding strength and improve ion conductivity. Furthermore, the optimization of mechanical properties, particularly through grafting lignin and CMC binders, is often required. Although most studies have been limited to a small number of charging/discharging cycles, promising results suggest that bio-based binder systems can withstand over 1000 cycles at sufficient C rates. These findings provide a great contribution to enabling a green transition of LIBs. Despite great progress, many challenges remain, including understanding the binder failure mechanisms during long-term testing, assessing the impact of an increased C rate on delamination and binder failure, optimizing the mechanical and ionic conductivity properties, investigating surface reactions contributing to the formation of an unstable solid electrolyte interface layer, and exploring the tunability of specific electrode design parameters such as density, porosity, and thickness. Moreover, the commercial use of bio-based systems remains currently limited due to an urgent need to increase the technological readiness levels and bringing these new solutions closer to industry. The performance level of these new binder systems needs to be optimized and balanced with binder resource availability, processability, and eventually, the overall production costs in comparison with currently used non-biobased binders.

## Figures and Tables

**Table 1:** Summary of the various applied bio-based binders and specific capacity performances reported for investigated anode and cathode materials in LIBs. The binder system is specified as “-based” since some chemical modifications were sometimes implemented, such as grafting and cross-linking. The specific capacity values are reported for the lowest C rate applied (usually 0.1 C) and when a stable performance was achieved.

Binder	Electrode	Specific Capacity (mAh g^−1^)	References
	**Anode:**		
CMC-based	Graphite	300	[60]
	Graphite	141	[37]
	Graphite	300	[70]
	Si-based	1153	[23]
	Si-based	1100	[64]
	Si-based	1000–1900	[65]
	Si-based	1544	[28]
CNF-based	Graphite	330	[77]
	Graphite	345	[73]
Lignin-based	Graphite	305	[47]
	Si-based	1914	[28]
	Si-based	939	[81]
	Si/graphite	492	[28]
Guar gum-based	Graphite	310	[84]
	Si-based	2222	[82]
	Si-based	1402	[39]
Xanthan gum	Graphite	250	[41]
	Si-based/graphene	725	[85]
Gum arabic	Graphite	200	[84]
	Si-based	2000	[30]
Okra gum	Si-based	1434	[40]
Carrageenan	Graphite	300	[84]
	Si-based	2031	[55]
Sodium alginate	Graphite	300	[84]
	Si/graphite	849	[49]
	Si-based	1000	[54]
Starch-based	Si-based	500	[46]
	Si-based	1864–2874	[46]
	Si-based	1100	[45]
	Si-based	2106	[50]
	Si-based	2000	[44]
	**Cathode:**		
Lignin	LiFePO_4_	148	[47]
	NMC111	140	[87]
CMC/lignin	NMC111	140	[88]
CMC	Li [Li_0.2_Mn_0.56_Ni_0.16_Co_0.08_]O_2_	230	[89]
Carboxymethyl chitosan/poly (ethylene oxide)	LiNi_0.5_Mn_1.5_O_4_	120	[90]
Tragacanth gum	LNMO-based	122	[43]
Sodium alginate	Li_2_TP	200	[48]

## Data Availability

Data sharing not applicable.

## References

[1] Winter M., Barnett B., Xu K. (2018). Before Li Ion Batteries. Chem. Rev..

[2] Xiang J., Wei Y., Zhong Y., Yang Y., Cheng H., Yuan L., Xu H., Huang Y. (2022). Building Practical High-Voltage Cathode Materials for Lithium-Ion Batteries. Adv. Mater..

[3] Tian Y., Zeng G., Rutt A., Shi T., Kim H., Wang J., Koettgen J., Sun Y., Ouyang B., Chen T. (2021). Promises and Challenges of Next-Generation “Beyond Li-Ion” Batteries for Electric Vehicles and Grid Decarbonization. Chem. Rev..

[4] Fukuda M., Iijima T., Collins D.H. (1975). Power Sources.

[5] Nakajima K. (1989). Conversation too Hot to Handle. Mainichi Daily News.

[6] Yazami R., Touzain P. (1983). A Reversible Graphite-Lithium Negative Electrode for Electrochemical Generators. J. Power Sources.

[7] Ozawa K. (1994). Lithium-Ion Rechargeable Batteries with LiCoO_2_ and Carbon Electrodes: The LiCoO_2_/C System. Solid State Ion..

[8] Ma Y., Ma J., Cui G. (2019). Small Things Make Big Deal: Powerful Binders of Lithium Batteries and Post-Lithium Batteries. Energy Storage Mater..

[9] Mishra A., Mehta A., Basu S., Malode S.J., Shetti N.P., Shukla S.S., Nadagouda M.N., Aminabhavi T.M. (2018). Electrode Materials for Lithium-Ion Batteries. Mater. Sci. Energy Technol..

[10] Sun Y., Liu N., Cui Y. (2016). Promises and Challenges of Nanomaterials for Lithium-Based Rechargeable Batteries. Nat. Energy.

[11] Nzereogu P.U., Omah A.D., Ezema F.I., Iwuoha E.I., Nwanya A.C. (2022). Anode Materials for Lithium-Ion Batteries: A Review. Appl. Surf. Sci. Adv..

[12] Lingappan N., Kong L., Pecht M. (2021). The Significance of Aqueous Binders in Lithium-Ion Batteries. Renew. Sustain. Energy Rev..

[13] Li J.-T., Wu Z.-Y., Lu Y.-Q., Zhou Y., Huang Q.-S., Huang L., Sun S.-G. (2017). Water Soluble Binder, an Electrochemical Performance Booster for Electrode Materials with High Energy Density. Adv. Energy Mater..

[14] Komaba S., Yabuuchi N., Ozeki T., Okushi K., Yui H., Konno K., Katayama Y., Miura T. (2010). Functional Binders for Reversible Lithium Intercalation into Graphite in Propylene Carbonate and Ionic Liquid Media. J. Power Sources.

[15] Nakazawa T., Ikoma A., Kido R., Ueno K., Dokko K., Watanabe M. (2016). Effects of Compatibility of Polymer Binders with Solvate Ionic Liquid Electrolytes on Discharge and Charge Reactions of Lithium-Sulfur Batteries. J. Power Sources.

[16] Tsao C.-H., Hsu C.-H., Kuo P.-L. (2016). Ionic Conducting and Surface Active Binder of Poly (Ethylene Oxide)-Block-Poly(Acrylonitrile) for High Power Lithium-Ion Battery. Electrochim. Acta.

[17] Komaba S., Okushi K., Ozeki T., Yui H., Katayama Y., Miura T., Saito T., Groult H. (2009). Polyacrylate Modifier for Graphite Anode of Lithium-Ion Batteries. Electrochem. Solid-State Lett..

[18] Pieczonka N.P.W., Borgel V., Ziv B., Leifer N., Dargel V., Aurbach D., Kim J.-H., Liu Z., Huang X., Krachkovskiy S.A. (2015). Lithium Polyacrylate (LiPAA) as an Advanced Binder and a Passivating Agent for High-Voltage Li-Ion Batteries. Adv. Energy Mater..

[19] Wang H., Sencadas V., Gao G., Gao H., Du A., Liu H., Guo Z. (2016). Strong Affinity of Polysulfide Intermediates to Multi-Functional Binder for Practical Application in Lithium–Sulfur Batteries. Nano Energy.

[20] Liu D., Zhao Y., Tan R., Tian L.-L., Liu Y., Chen H., Pan F. (2017). Novel Conductive Binder for High-Performance Silicon Anodes in Lithium Ion Batteries. Nano Energy.

[21] Drofenik J., Gaberscek M., Dominko R., Poulsen F.W., Mogensen M., Pejovnik S., Jamnik J. (2003). Cellulose as a Binding Material in Graphitic Anodes for Li Ion Batteries: A Performance and Degradation Study. Electrochim. Acta.

[22] Luo L., Xu Y., Zhang H., Han X., Dong H., Xu X., Chen C., Zhang Y., Lin J. (2016). Comprehensive Understanding of High Polar Polyacrylonitrile as an Effective Binder for Li-Ion Battery Nano-Si Anodes. ACS Appl. Mater. Interfaces.

[23] Munao D., van Erven J.W.M., Valvo M., Garcia-Tamayo E., Kelder E.M. (2011). Role of the Binder on the Failure Mechanism of Si Nano-Composite Electrodes for Li-Ion Batteries. J. Power Sources.

[24] Chen H., Ling M., Hencz L., Ling H.Y., Li G., Lin Z., Liu G., Zhang S. (2018). Exploring Chemical, Mechanical, and Electrical Functionalities of Binders for Advanced Energy-Storage Devices. Chem. Rev..

[25] Zou F., Manthiram A. (2020). A Review of the Design of Advanced Binders for High-Performance Batteries. Adv. Energy Mater..

[26] Cholewinski A., Si P., Uceda M., Pope M., Zhao B. (2021). Polymer Binders: Characterization and Development toward Aqueous Electrode Fabrication for Sustainability. Polymers.

[27] Kwon T., Choi J.W., Coskun A. (2018). The Emerging Era of Supramolecular Polymeric Binders in Silicon Anodes. Chem. Soc. Rev..

[28] Luo C., Du L., Wu W., Xu H., Zhang G., Li S., Wang C., Lu Z., Deng Y. (2018). Novel Lignin-Derived Water-Soluble Binder for Micro Silicon Anode in Lithium-Ion Batteries. ACS Sustain. Chem. Eng..

[29] Wu H., Cui Y. (2012). Designing Nanostructured Si Anodes for High Energy Lithium Ion Batteries. Nano Today.

[30] Ling M., Xu Y., Zhao H., Gu X., Qiu J., Li S., Wu M., Song X., Yan C., Liu G. (2015). Dual-Functional Gum Arabic Binder for Silicon Anodes in Lithium Ion Batteries. Nano Energy.

[31] Jabbour L., Bongiovanni R., Chaussy D., Gerbaldi C., Beneventi D. (2013). Cellulose-Based Li-Ion Batteries: A Review. Cellulose.

[32] Liedel C. (2020). Sustainable Battery Materials from Biomass. ChemSusChem.

[33] Wang X., Yao C., Wang F., Li Z. (2017). Cellulose-Based Nanomaterials for Energy Applications. Small.

[34] Buqa H., Holzapfel M., Krumeich F., Veit C., Novák P. (2006). Study of Styrene Butadiene Rubber and Sodium Methyl Cellulose as Binder for Negative Electrodes in Lithium-Ion Batteries. J. Power Sources.

[35] Chang W.J., Lee G.H., Cheon Y.J., Kim J.T., Lee S.I., Kim J., Kim M., Park W.I., Lee Y.J. (2019). Direct Observation of Carboxymethyl Cellulose and Styrene–Butadiene Rubber Binder Distribution in Practical Graphite Anodes for Li-Ion Batteries. ACS Appl. Mater. Interfaces.

[36] Nirmale T.C., Kale B.B., Varma A.J. (2017). A Review on Cellulose and Lignin Based Binders and Electrodes: Small Steps towards a Sustainable Lithium Ion Battery. Int. J. Biol. Macromol..

[37] Li W.-C., Lin C.-H., Ho C.-C., Cheng T.-T., Wang P.-H., Wen T.-C. (2022). Superior Performances of Supercapacitors and Lithium-Ion Batteries with Carboxymethyl Cellulose Bearing Zwitterions as Binders. J. Taiwan Inst. Chem. Eng..

[38] Carvalho D.V., Loeffler N., Hekmatfar M., Moretti A., Kim G.-T., Passerini S. (2018). Evaluation of Guar Gum-Based Biopolymers as Binders for Lithium-Ion Batteries Electrodes. Electrochim. Acta.

[39] Jia M., Qin X., Zhang X., Wang J., Liu S., Wang L., Zhang Z., Miao N., Jiang G., Li Y. (2023). Novel Rigid-Flexible Hydrogenated Carboxyl Nitrile Rubber-Guar Gum Binder for Ultra-Long Cycle Silicon Anodes in Lithium-Ion Batteries. J. Power Sources.

[40] Ling H.Y., Hencz L., Chen H., Wu Z., Su Z., Chen S., Yan C., Lai C., Liu X., Zhang S. (2021). Sustainable Okra Gum for Silicon Anode in Lithium-Ion Batteries. Sustain. Mater. Technol..

[41] Wang Z., Dang G., Zhang Q., Xie J. (2017). Xanthan Gum as a Potential Binder for Graphite Anode in Lithium-Ion Batteries. Int. J. Electrochem. Sci..

[42] Versaci D., Nasi R., Zubair U., Amici J., Sgroi M., Dumitrescu M.A., Francia C., Bodoardo S., Penazzi N. (2017). New Eco-Friendly Low-Cost Binders for Li-Ion Anodes. J. Solid State Electrochem..

[43] Versaci D., Apostu O.D., Dessantis D., Amici J., Francia C., Minella M., Bodoardo S. (2023). Tragacanth, an Exudate Gum as Suitable Aqueous Binder for High Voltage Cathode Material. Batteries.

[44] Bie Y., Yang J., Nuli Y., Wang J. (2016). Oxidized Starch as a Superior Binder for Silicon Anodes in Lithium-Ion Batteries. RSC Adv..

[45] Hapuarachchi S.N.S., Wasalathilake K.C., Nerkar J.Y., Jaatinen E., O’Mullane A.P., Yan C. (2020). Mechanically Robust Tapioca Starch Composite Binder with Improved Ionic Conductivity for Sustainable Lithium-Ion Batteries. ACS Sustain. Chem. Eng..

[46] Jin B., Wang D., Song L., Cai Y., Ali A., Hou Y., Chen J., Zhang Q., Zhan X. (2021). Biomass-Derived Fluorinated Corn Starch Emulsion as Binder for Silicon and Silicon Oxide Based Anodes in Lithium-Ion Batteries. Electrochim. Acta.

[47] Lu H., Cornell A., Alvarado F., Behm M., Leijonmarck S., Li J., Tomani P., Lindbergh G. (2016). Lignin as a Binder Material for Eco-Friendly Li-Ion Batteries. Materials.

[48] Zhang S., Ren S., Han D., Xiao M., Wang S., Meng Y. (2019). Aqueous Sodium Alginate as Binder: Dramatically Improving the Performance of Dilithium Terephthalate-Based Organic Lithium Ion Batteries. J. Power Sources.

[49] Gendensuren B., Oh E.-S. (2018). Dual-Crosslinked Network Binder of Alginate with Polyacrylamide for Silicon/Graphite Anodes of Lithium Ion Battery. J. Power Sources.

[50] Huang J., Liu B., Zhang P., Li R., Zhou M., Wen B., Xia Y., Okada S. (2021). A Low-Cost and Sustainable Cross-Linked Dextrin as Aqueous Binder for Silicon Anodes in Lithium-Ion Batteries. Solid State Ion..

[51] Kumagai S., Abe Y., Tomioka M., Kabir M. (2021). Suitable Binder for Li-Ion Battery Anode Produced from Rice Husk. Sci. Rep..

[52] Li L., Li T., Sha Y., Zhang C., Ren B., Zhang L., Zhang S. (2021). H-Bond Network-Regulated Binder for Si/Graphite Anodes. Ind. Eng. Chem. Res..

[53] Wang H., Wei D., Zhang B., Ji Z., Wang L., Ling M., Liang C. (2021). Epoxy Cross-Linking Enhanced the Toughness of Polysaccharides as a Silicon Anode Binder for Lithium-Ion Batteries. ACS Appl. Mater. Interfaces.

[54] Wang Z., Huang T., Yu A. (2021). A Carboxymethyl Vegetable Gum as a Robust Water Soluble Binder for Silicon Anodes in Lithium-Ion Batteries. J. Power Sources.

[55] Jang W., Rajeev K.K., Thorat G.M., Kim S., Kang Y., Kim T.-H. (2022). Lambda Carrageenan as a Water-Soluble Binder for Silicon Anodes in Lithium-Ion Batteries. ACS Sustain. Chem. Eng..

[56] Chang Y.-L., Sharma S.U., Shiu J.-P., Lee J.-T. (2021). Xylitol-Maleic Anhydride as Small-Molecule Binders for Silicon Anodes in Lithium-Ion Batteries. J. Electrochem. Soc..

[57] Kasnatscheew J., Evertz M., Streipert B., Wagner R., Klöpsch R., Vortmann B., Hahn H., Nowak S., Amereller M., Gentschev A.-C. (2016). The Truth about the 1st Cycle Coulombic Efficiency of LiNi_1/3_Co_1/3_Mn_1/3_O_2_ (NCM) Cathodes. Phys. Chem. Chem. Phys..

[58] Sarkar A., Manohar C.V., Mitra S. (2020). A Simple Approach to Minimize the First Cycle Irreversible Loss of Sodium Titanate Anode towards the Development of Sodium-Ion Battery. Nano Energy.

[59] Memarzadeh Lotfabad E., Kalisvaart P., Kohandehghan A., Karpuzov D., Mitlin D. (2014). Origin of Non-SEI Related Coulombic Efficiency Loss in Carbons Tested against Na and Li. J. Mater. Chem. A.

[60] Shin D., Park H., Paik U. (2017). Cross-Linked Poly(Acrylic Acid)-Carboxymethyl Cellulose and Styrene-Butadiene Rubber as an Efficient Binder System and Its Physicochemical Effects on a High Energy Density Graphite Anode for Li-Ion Batteries. Electrochem. Commun..

[61] Ui K., Kikuchi S., Mikami F., Kadoma Y., Kumagai N. (2007). Improvement of Electrochemical Characteristics of Natural Graphite Negative Electrode Coated with Polyacrylic Acid in Pure Propylene Carbonate Electrolyte. J. Power Sources.

[62] Nordh T., Jeschull F., Younesi R., Koçak T., Tengstedt C., Edström K., Brandell D. (2017). Different Shades of Li_4_Ti_5_O_12_ Composites: The Impact of the Binder on Interface Layer Formation. ChemElectroChem.

[63] Lee J.-H., Lee S., Paik U., Choi Y.-M. (2005). Aqueous Processing of Natural Graphite Particulates for Lithium-Ion Battery Anodes and Their Electrochemical Performance. J. Power Sources.

[64] Li J., Lewis R.B., Dahn J.R. (2006). Sodium Carboxymethyl Cellulose: A Potential Binder for Si Negative Electrodes for Li-Ion Batteries. Electrochem. Solid-State Lett..

[65] Karkar Z., Guyomard D., Roué L., Lestriez B. (2017). A Comparative Study of Polyacrylic Acid (PAA) and Carboxymethyl Cellulose (CMC) Binders for Si-Based Electrodes. Electrochim. Acta.

[66] Qiu L., Shao Z., Liu M., Wang J., Li P., Zhao M. (2014). Synthesis and Electrospinning Carboxymethyl Cellulose Lithium (CMC-Li) Modified 9,10-Anthraquinone (AQ) High-Rate Lithium-Ion Battery. Carbohydr. Polym..

[67] Melnick R.L., Huff J., Chou B.J., Miller R.A. (1990). Carcinogenicity of 1,3-Butadiene in C57BL/6 × C3H F1 Mice at Low Exposure Concentrations. Cancer Res..

[68] Park J.H., Kim S.H., Ahn K.H. (2023). Role of Carboxymethyl Cellulose Binder and Its Effect on the Preparation Process of Anode Slurries for Li-Ion Batteries. Colloids Surf. Physicochem. Eng. Asp..

[69] García A., Culebras M., Collins M.N., Leahy J.J. (2018). Stability and Rheological Study of Sodium Carboxymethyl Cellulose and Alginate Suspensions as Binders for Lithium Ion Batteries. J. Appl. Polym. Sci..

[70] Jeschull F., Lacey M.J., Brandell D. (2015). Functional Binders as Graphite Exfoliation Suppressants in Aggressive Electrolytes for Lithium-Ion Batteries. Electrochim. Acta.

[71] Park H., Lee D., Song T. (2018). Synthesis of Carboxymethyl Cellulose Lithium by Weak Acid Treatment and Its Application in High Energy-Density Graphite Anode for Li-Ion Batteries. Ind. Eng. Chem. Res..

[72] Kil K.C., Paik U. (2015). Lithium Salt of Carboxymethyl Cellulose as an Aqueous Binder for Thick Graphite Electrode in Lithium Ion Batteries. Macromol. Res..

[73] Lu H., Behm M., Leijonmarck S., Lindbergh G., Cornell A. (2016). Flexible Paper Electrodes for Li-Ion Batteries Using Low Amount of TEMPO-Oxidized Cellulose Nanofibrils as Binder. ACS Appl. Mater. Interfaces.

[74] Jabbour L., Gerbaldi C., Chaussy D., Zeno E., Bodoardo S., Beneventi D. (2010). Microfibrillated Cellulose–Graphite Nanocomposites for Highly Flexible Paper-like Li-Ion Battery Electrodes. J. Mater. Chem..

[75] Phillips T.K., Bhinde T., Clarke S.M., Lee S.Y., Mali K.S., Feyter S.D. (2010). Adsorption of Aldehydes on a Graphite Substrate: Combined Thermodynamic Study of C6−C13 Homologues with a Structural and Dynamical Study of Dodecanal. J. Phys. Chem. C.

[76] Françon H.S., Gorur Y.C., Montanari C., Larsson P.A., Wågberg L. (2022). Toward Li-Ion Graphite Anodes with Enhanced Mechanical and Electrochemical Properties Using Binders from Chemically Modified Cellulose Fibers. ACS Appl. Energy Mater..

[77] Gorur Y.C., Francon H.S., Sethi J., Maddalena L., Montanari C., Reid M.S., Erlandsson J., Carosio F., Larsson P.A., Wågberg L. (2022). Rapidly Prepared Nanocellulose Hybrids as Gas Barrier, Flame Retardant, and Energy Storage Materials. ACS Appl. Nano Mater..

[78] Chen T., Zhang Q., Pan J., Xu J., Liu Y., Al-Shroofy M., Cheng Y.-T. (2016). Low-Temperature Treated Lignin as Both Binder and Conductive Additive for Silicon Nanoparticle Composite Electrodes in Lithium-Ion Batteries. ACS Appl. Mater. Interfaces.

[79] Jung H.Y., Lee J.S., Han H.T., Jung J., Eom K., Lee J.T. (2022). Lignin-Based Materials for Sustainable Rechargeable Batteries. Polymers.

[80] Moreno A., Morsali M., Sipponen M.H. (2021). Catalyst-Free Synthesis of Lignin Vitrimers with Tunable Mechanical Properties: Circular Polymers and Recoverable Adhesives. ACS Appl. Mater. Interfaces.

[81] Yuan J.-M., Ren W.-F., Wang K., Su T.-T., Jiao G.-J., Shao C.-Y., Xiao L.-P., Sun R.-C. (2022). Ultrahighly Elastic Lignin-Based Copolymers as an Effective Binder for Silicon Anodes of Lithium-Ion Batteries. ACS Sustain. Chem. Eng..

[82] Liu J., Zhang Q., Zhang T., Li J.-T., Huang L., Sun S.-G. (2015). A Robust Ion-Conductive Biopolymer as a Binder for Si Anodes of Lithium-Ion Batteries. Adv. Funct. Mater..

[83] Schlemmer W., Selinger J., Hobisch M.A., Spirk S. (2021). Polysaccharides for Sustainable Energy Storage—A Review. Carbohydr. Polym..

[84] Cuesta N., Ramos A., Cameán I., Antuña C., García A.B. (2015). Hydrocolloids as Binders for Graphite Anodes of Lithium-Ion Batteries. Electrochim. Acta.

[85] Chen D., Yi R., Chen S., Xu T., Gordin M.L., Wang D. (2014). Facile Synthesis of Graphene–Silicon Nanocomposites with an Advanced Binder for High-Performance Lithium-Ion Battery Anodes. Solid State Ion..

[86] He C., Gendensuren B., Kim H., Lee H., Oh E.-S. (2020). Electrochemical Performance of Polysaccharides Modified by the Introduction of SO_3_H as Binder for High-Powered Li_4_Ti_5_O_12_ Anodes in Lithium-Ion Batteries. J. Electroanal. Chem..

[87] Bryntesen S.N., Finne P.H., Svensson A.M., Shearing P.R., Tolstik N., Sorokina I.T., Vinje J., Lamb J.J., Burheim O.S. (2023). Structured Aqueous Processed Lignin-Based NMC Cathodes for Energy-Dense LIBs with Improved Rate Capability. J. Mater. Chem. A.

[88] Bryntesen S.N., Tolstorebrov I., Svensson A.M., Shearing P., Lamb J.J., Burheim O.S. (2023). Introducing Lignin as a Binder Material for the Aqueous Production of NMC111 Cathodes for Li-Ion Batteries. Mater. Adv..

[89] Li J., Klöpsch R., Nowak S., Kunze M., Winter M., Passerini S. (2011). Investigations on Cellulose-Based High Voltage Composite Cathodes for Lithium Ion Batteries. J. Power Sources.

[90] Zhong H., Lu J., He A., Sun M., He J., Zhang L. (2017). Carboxymethyl Chitosan/Poly(Ethylene Oxide) Water Soluble Binder: Challenging Application for 5V LiNi_0.5_Mn_1.5_O_4_ Cathode. J. Mater. Sci. Technol..

[91] Kim U.-H., Kuo L.-Y., Kaghazchi P., Yoon C.S., Sun Y.-K. (2019). Quaternary Layered Ni-Rich NCMA Cathode for Lithium-Ion Batteries. ACS Energy Lett..

[92] Wang Z., Dupré N., Gaillot A.-C., Lestriez B., Martin J.-F., Daniel L., Patoux S., Guyomard D. (2012). CMC as a Binder in LiNi0.4Mn1.6O4 5V Cathodes and Their Electrochemical Performance for Li-Ion Batteries. Electrochim. Acta.

[93] Zhao T., Meng Y., Ji R., Wu F., Li L., Chen R. (2019). Maintaining Structure and Voltage Stability of Li-Rich Cathode Materials by Green Water-Soluble Binders Containing Na+ Ions. J. Alloys Compd..

[94] Xu J., Li K., Liu L., Ma J., Zhang H. (2023). Tannic Acid—A Bridge and Suspending Agent for Lithium Cobalt Oxide and Reduced Graphene Oxide: A Lodestar for Lithium-Ion Batteries. Environ. Technol..

[95] Porcher W., Lestriez B., Jouanneau S., Guyomard D. (2008). Design of Aqueous Processed Thick LiFePO_4_ Composite Electrodes for High-Energy Lithium Battery. J. Electrochem. Soc..

[96] Porcher W., Moreau P., Lestriez B., Jouanneau S., Guyomard D. (2007). Is LiFePO_4_ Stable in Water?: Toward Greener Li–Ion Batteries. Electrochem. Solid-State Lett..

